# Determinants of maternity waiting home utilization in Sidama Zone, Southern Ethiopia: A cross-sectional study

**DOI:** 10.1371/journal.pone.0264416

**Published:** 2022-03-14

**Authors:** Zelalem Tenaw, Rekiku Fikre, Hirut Gemeda, Ayalew Astatkie

**Affiliations:** 1 Department of Midwifery, College of Medicine and Health Sciences, Hawassa University, Hawassa, Ethiopia; 2 School of Public Health, College of Medicine and Health Sciences, Hawassa University, Hawassa, Ethiopia; University of Abuja/University of Abuja Teaching Hospital, NIGERIA

## Abstract

**Objective:**

To estimate the magnitude of maternity waiting home utilization and identify its associated factors in Sidama Zone, Southern Ethiopia.

**Methods:**

A community-based cross-sectional study was conducted on a total of 748 mothers who gave birth within the last year in the selected woredas (districts) of Sidama Zone. Data were collected from April 1–30, 2019 by using pre-tested and structured questionnaires. Data were coded and entered into EpiData version 3.5.1 and exported to Stata Version 13 software for analysis. Multivariable logistic regression analysis was performed to identify factors associated with maternity waiting home utilization adjusting for confounders.

**Results:**

The mean (SD) of the age of the mothers was 31.26(6.42). Utilization of maternity waiting home in Sidama Zone was 67.25% (95% CI: 63.79–70.53).Maternity waiting home utilization was positively associated with protestant religion (AOR = 1.7; 95% CI: 1.00–2.82) and having a spouse who can read and write (AOR = 2.0; 95%CI: 1.11–3.66) while it was negatively associated with maternal age of 31–40 (AOR = 0.4; 95%CI: 0.28–0.64) relative to the age group of 20–30, daily laborer occupation of mothers (AOR = 0.2; 95%CI: 0.06–0.76), monthly income under the poverty level (825-1320EBR) (AOR = 0.6; 95%CI: 0.36–0.92) relative with extreme poverty line (<825 EBR), lack of knowledge about maternity waiting home (AOR = 0.009; 95%CI: 0.002–0.03).

**Conclusions:**

Women who had knowledge about maternity waiting home, had a husband who can read and write and protestant religion followers have higher probabilities of maternity waiting home utilization, whereas women (31–40 years old), daily laborers and whose family income is below the poverty level have lower probabilities of maternity waiting home utilization. Therefore, Health education about maternity waiting home utilization, spouse education, and women’s economic empowerment is crucial to enhance maternity waiting home utilization.

## Introduction

Maternity waiting home (MWH) is a health facility residential accommodation of pregnant mothers starting from their term period of pregnancy. It is an intervention designed to improve access to skilled deliveries in low-income countries **[1, 2]**.

In low-income countries like Ethiopia maternal morbidity and mortality is high **[1, 3].** The utilization of a maternity waiting home is a proven strategy to decrease maternal mortality and stillbirth rate **[4–6].** Access to comprehensive emergency obstetric care is limited in Ethiopia. MWHs are part of the strategies utilized to improve access to too hard-to-reach rural populations **[4, 7]**. A skilled birth attendant is pivotal for decreasing maternal and neonatal mortality, however many women in low and middle-income countries gave birth at home without skilled birth attendants help **[1, 8–11].** In developing countries like Ethiopia, 30% of maternal mortality is due to a lack of skilled delivery services **[12, 13]. According to EDHS, 2016 report still the coverage of skilled and institutional delivery in the south nation’s nationalities and peoples’ region were only 28.6% and 25.5% respectively [14].** Lower rates of maternal and perinatal death were reported from communities with maternity waiting homes compared with those without maternity waiting homes **[12, 15–17]**.

Different factors affect the utilization of MWHs in Africa; A study in Ghana and Zambia reported that women could only use facility-based delivery services if they obtained permission from their husbands **[18, 19].** A study from Kenya reports only 28% of women knew of the existence of the MWH and the majority (95%) reported that they would require their husband’s permission to use it **[20].**

Despite the long years of existence of this service in Ethiopia, the practice has not been adequately assessed so far **[4, 21].** Therefore, this study aimed to estimate the magnitude of maternity waiting home utilization and its associated factors in Sidama Zone, Southern Ethiopia.

## Methods and materials

### Study design and settings

A Community based cross-sectional study was carried out from April 1-30/2019 among mothers who gave birth in the last one year in the Sidama zone. Sidama Zone is one of the Zones found in the Southern Nations Nationalities and Peoples Regional State (SNNPRS) of Ethiopia.

### Sample size and sampling procedure

There are twenty woredas (districts) and two city administrations in the zone. According to the Sidama Zone Health Department, the total population in 2014/2015 was projected to be 3,676,576 **[22]**. There are seven governmental hospitals, 148 governmental health centers, and 524 health posts in the zone. Regarding human resources for health, the zone had 1857 obstetrics care providers (Physicians, Midwives, Public health officers, and nurses).

The sample size was determined using the software Epi Info version 7 with the following assumptions: 95% confidence interval with 28.18% prevalence of maternity waiting home utilization **[16],** with a level of confidence (α) of 0.05, and 5% margin of error **(d = 0.05).** The sample size for associated factors of maternity waiting home utilization was also calculated considering a confidence level of 95%, power of 80%, a ratio of unexposed-to-exposed of 1, and taking various factors. Then the largest of the calculated sample sizes was taken as a final sample size. Accordingly, distance to the health facility was considered as a factor to utilize maternity waiting homes **[1],** which yielded a sample size of 340. By considering a design effect of 2 for two-stage sampling, the total sample size was 680. After adjusting for an anticipated 10% nonresponse rate, the final sample size was 748.

The sample size was proportionally allocated for selected woredas and kebeles based on the number of mothers who gave birth within the last year based on a census conducted before the actual study. To select the study participants a simple random sampling procedure was implemented.

### Data collection procedures

The data were collected through face-to-face interviews by using structured and pretested questionnaires. The questionnaire was prepared by reviewing existing literature, which consists of sociodemographic characteristics, personal characteristics, and obstetric history. The pretest was done on 5% of the sample among mothers with similar characteristics to those included in the study.

Ten (10) obstetrics care providers who have a diploma in midwifery and who were proficient in the local language (Sidamu Afoo) were recruited for data collection. The training was given for two days on the objective, relevance of the study, confidentiality of information, respondents’ rights, informed consent, and technique of interview; two midwives with a Bachelor’s degree were trained and supervised the data collection.

### Data analysis

Data entry was done using EpiData 3.5.1 and exported to Stata version 13 software for analysis. After exporting the data to stata, data cleaning was done. Then descriptive analysis was done for the data to calculate the frequency, proportion, and distribution. The logistic regression model was utilized to analyze the data. First binary logistic regression was done to check the presence of associations and then the independent variables that had P-value less than or equal to 0.25 will be considered as eligible for multivariable analysis. Then multivariable logistic regression was conducted to check the presence of an association between independent variables and MWH utilization. Adjusted odds ratios with 95% confidence interval were used to decide whether a significant association exists and its strength.

### Ethics statement

Ethical clearance was obtained from the Institutional Review Board at the College of Medicine and Health Sciences of Hawassa University. Sidama Zone Health Office and management of the respective woreda health offices offered consent to conduct the study. Written consent was gained from the study participants before data collection started. Anonymous questionnaires were used to assure the confidentiality of study participants.

## Results

### Socio-demographic characteristic of study participants

A total of 748 mothers participated in the study, with a 99.59% response rate. The ages of the participants ranged from 20 to 61 years with a mean (±standard deviation) age of 31.26 (±6.42) years **(Table 1).**

**Table 1:** Socio-demographic and economic characteristics of study participants in Sidama Zone, southern Ethiopia, April 2019 (n = 745).

Variables		Frequency	Percentage
**Age(years)**	20–30	441	59.19
	31–40	245	32.89
	41–50	56	7.52
	51–61	3	0.40
**Religion**	Orthodox	97	13.02
	Muslim	89	11.95
	Protestant	527	70.74
	Catholic	32	4.3
**Marital status**	Married	692	92.89
	Single	12	1.61
	Divorced	11	1.48
	Widowed	30	4.03
**Occupation of the mother**	Housewife	614	82.42
	Government employed	12	1.62
	Private employed	85	11.41
	NGO employed	4	0.54
	student	12	1.61
	Daily labor	14	1.88
	Other^ 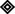 ^	4	0.54
**Occupation of the spouse**	Farmer	388	52.08
	Government employed	19	2.55
	Private employed	152	33.83
	student	8	1.07
	Daily labor	69	9.26
	Other^≥^	9	1.21
**Monthly income**	<825(extreme poverty)	557	74.77
	825-1320(under poverty)	115	15.44
	>1320(above poverty)	73	9.80
**Family size**	2–3	150	20.13
	4–6	448	60.13
	7–10	147	19.73
**Educational status of the mother**	Illiterate	292	39.19
	Read and write	195	26.17
	Primary school complete	188	25.23
	Secondary school complete	50	6.71
	Graduated from collage/university	20	2.68
**Educational status of the spouse**	Illiterate	209	28.05
	Read and write	223	29.93
	Primary school complete	204	27.38
	Secondary school complete	82	11.01
	Graduated from collage/university	27	3.62

Other^Ω:^ Merchant, Shop keeper other^
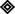
^: Fisherman, shop keeper.

### Maternity waiting home utilization

**Fig 1:**
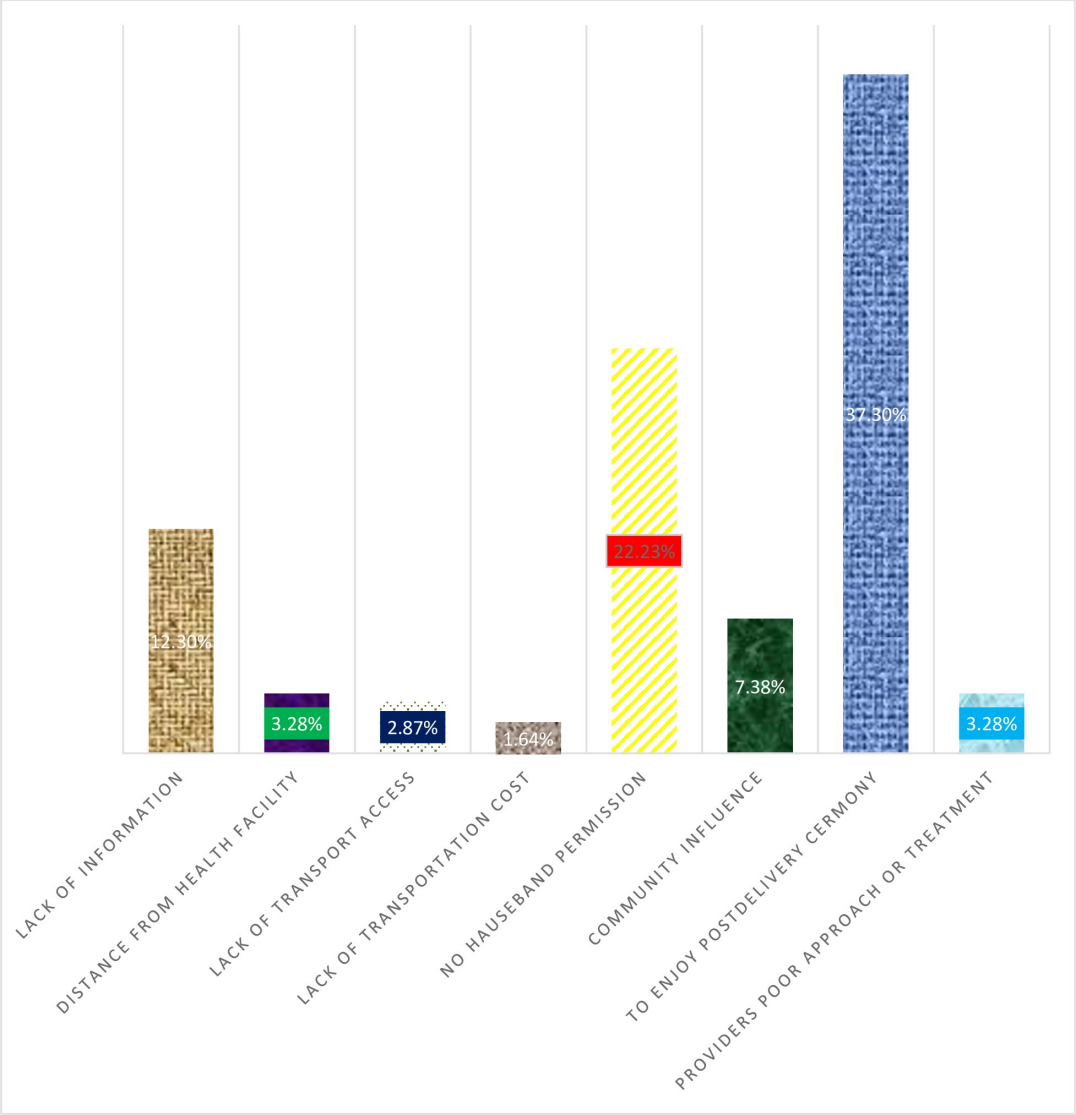
Complained reasons of mother for not utilizing maternity waiting home.

In Sidama Zone the prevalence of maternity waiting home utilization is 67.25% (95% CI: 63.79%-70.53%) (n = 501). As listed in Fig 1, there are different reasons mentioned for not utilizing the maternity waiting home. The most common reason is to enjoy a postnatal ceremony at home in the presence of the family members (37.30%; n = 91) **(See Fig 1).**

### Factors associated with maternity waiting home utilization

Maternal age of 31–40 years (AOR = 0.4; 95%CI: 0.28–0.64)relative to age group 20–30, being a daily laborer by occupation (AOR = 0.2; 95%CI: 0.06–0.76), protestant religion (AOR = 1.7; 95%CI: 1.00–2.82), under poverty monthly income(825–1320 EBR) (AOR = 0.6; 95%CI: 0.36–0.92) relative with extreme poverty(<825 EBR), lack of knowledge about MWH (AOR = 0.009, 95%CI: 0.002–0.03) and spouse who can read and write (AOR = 2.0; 95%CI: 1.11–3.66) relative to illiterate spouses were significantly associated with maternity waiting home utilization (**Table 2**).

**Table 2:** Logistic regression analysis results of participants for maternity waiting home utilization in Sidama Zone, southern Ethiopia, April/2019 (n = 745).

Characteristics		Utilize MWH		OR (95%CI)	
		Yes	No	Crude	Adjusted
Age	20–30	72.11	27.89	1.00	
	31–40	56.73%	43.27	**0.5(0.37-.706)** *	**0.4(.28–0.64)** **
	41–50	78.57	21.43	1.4(.72–2.77)*	
	51–61	0	100%		
Marital status	Married	67.63	32.37	1.00	
	Single	33.33	66.67	**0.2(0.07–0.80)** *	
	Divorced	72.73	27.27	1.3(0.33–4.85)	
	Widowed	70.00	30.00	1.1(0.50–2.47)	
Religion	Orthodox	57.73	42.27	1.00	
	Muslim	58.43	41.57	1.0(0.57–1.84)	
	Protestant	70.97	29.03	**1.8(1.14 2.79)** *	**1.7(1.00–2.82)** **
	Catholic	59.38	40.63	1.0(0.47–2.41)	
Occupation of the mother	House wife	68.40	31.60	1.00	
	Government employed	66.67	33.33	0.9(0.27–3.10)	
	Private employed	62.35	37.65	0.8(0.47–1.22)	
	NGO employed	0	100		
	Student	100	0		
	Daily laborer	28.57	71.43	**0.2(0.05–0.59)** *	**0.2(0.06–0.76)** **
	Other^Ω^	100	0		
Occupation of the spouse	Farmer	67.78	32.22	1.00	
	Government employed	100	0		
	Private employed	63.10	36.90	0.8(0.58–1.13)	
	Student	100	0		
	Daily laborer	66.67	33.33	0.9(0.55–1.63)	
	Other^ 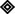 ^	66.67	33.33	0.9(0.23 3.86)	
Family monthly income	<825(Extreme poverty)	68.22	31.78	1.00	
	825-1320(Under poverty)	59.13	40.87	0.6(0.44–1.02)	**0.6(0.36–0.92)** **
	>1320(Above poverty)	72.60	27.40	1.2(0.71–2.12)	
Family size	2–3	68.00	32.00	1.00	
	4–6	69.87	30.13	1.0(0.73–1.62)	
	7–10	58.50	41.50	0.6(0.41–1.06)	
Educational status of the mother	Illiterate	61.30	38.70	1.00	
	Read and write	71.28	28.72	**1.6(1.06–2.31)** *	
	Primary school complete	72.87	27.13	**1.7(1.14–2.53)** *	
	Secondary school complete	52.00	48.00	0.6(0.37–1.25)	
	Graduated from collage/university	100	0		
Knowledge about Mwhu	Yes	98.20	0.60	1.00	
	No	65.98	34.02	**0.07(0.04–0.13)** *	**0.009(0.002–0.03)** **
Educational status of spouse	Illiterate	61.72	38.28	1.00	
	Read and write	77.13	22.87	**2.1(1.38–3.18)** *	**2.0(1.11–3.662)** **
	Primary school complete	61.76	38.24	1.0(0.67–1.48)	
	Secondary school complete	67.07	32.93	1.2(0.74–2.16)	
	Graduated from collage/university	70.37	29.63		
Gravidity	Primigravida	78.68	21.32	1.00	
	Multigravida	64.82	35.18	**0.5(0.32–0.78)** *	
	Grand multigravida	63.95	36.05	**0.5(0.26–0.88)** *	
Parity	Primipara	75.84	24.16	**1.00**	
	Multipara	65.43	34.57	**0.6(0.39–0.91)** *	
	Grand multipara	63.10	36.90	**0.5(0.30–0.97)** *	

*P-value <0.05,

** P-value < 0.05 after adjustment for socio demographic characteristics and some concepts of maternity waiting home utilization.

## Discussion

The prevalence of maternity waiting home utilization in the Sidama Zone is 67.25%. This finding is higher than the finding reported in Gurage (50%) **[23],** Bench Maji (39%) **[24]** and Jima Zone (7%) **[25]**. The reason for the difference might be the difference in the study participants and study period. The Jimma Zone study was done among 3784 women who were selected from purposively selected districts that have high population density **[25].**

Mothers in the age group of 31–40 years had 60% lower odds of maternity waiting home utilization compared to mothers in the age group of 20–30 years. This might be because women in this age group may have experienced several home births and therefore reluctant to patronize maternity waiting homes. Women who were protestant religious followers were more likely to utilize maternity waiting (AOR = 1.7). The possible reason might be the religious doctrine which emphasizes sensitive and current issues.

Women who were daily laborers had 80% lesser odds of utilizing maternity waiting homes. This may be because women in this group may likely continue daily work until there due dates of delivery so as to be able to earn a living and provide economic support for their families. Similarly, women whose family income is under the poverty threshold were less likely to utilize maternity waiting homes (AOR = 0.6). This evidence is consistent with the study conducted in Malawi [26].

Women who had a lack of knowledge on maternity waiting homes were also less likely to utilize the maternity waiting home (AOR = 0.009). This evidence is supported by the study conducted in 2017 among low and middle-income countries **[27].** The association between lack of knowledge about MWHs and their utilization is not surprising as women who do not know about their existence may also not have knowledge about their beneficial effect and where they are situated and hence unlikely to utilize the facilities.

Women who had a husband who can read and write were more likely to utilize maternity waiting homes (AOR = 2.0). This evidence is consistent with the study conducted in Guragie Zone (AOR = 5.4) **[28]**. Husbands who are literate are more likely to support utilization of health care services including MWHs. This is consistent with findings from a previous study that evaluated the effect of husband’s education on utilization of maternity services by their wives **[29]**.

This was a community-based study which involved women from all the districts and therefore reflects a true situation regarding the utilization of maternity waiting homes in the zone. However, the cross-sectional study design may have limited exploration of community -level determinants of utilization of maternity waiting homes in Sidama Zone, Southern Ethiopia.

## Conclusion

Women who had knowledge about maternity waiting home, had a husband who can read and write, and are protestant religion followers have increased probabilities of maternity waiting home utilization, whereas maternal age of 31–40 years, who were daily laborers and whose family income is under poverty level had decreased probabilities of maternity waiting home utilization. Therefore, health education and counseling about maternity waiting home utilization, spouse education, and women’s economic empowerment are crucial to enhance maternity waiting home utilization.
